# Supplementary material for: Are carbon emissions trading and green financial instruments synergistic? -Comprehensive quantitative research based on content analysis
Source: PLoS One. 2024 Mar 7;19(3):e0298601. doi: 10.1371/journal.pone.0298601 (PMC10919601; doi:10.1371/journal.pone.0298601)
Supplement: S1 Data — (ZIP) [file pone.0298601.s001.zip › Data/地级市Z_F工作报告.zip!/║╙─╧/╫ñ┬φ╡Ω╩╨/└┤╫╘╣1⁄2╓┌║┼í╛┬φ┐╦╩2╛▌═°í┐_2007.docx]

各位代表:

现在，我代表市人民政府，向大会作工作报告，请予审议，并请各位政协委员提出意见。

一、过去六年的工作回顾

2001年以来，市一届人民政府在省委、省政府和市委的正确领导下，在市人大、市政协的监督支持下，认真贯彻"三个代表"重要思想，全面落实科学发展观，切实履行人民赋予的职责，经过全市上下的共同努力，改革开放和现代化建设取得了显著成就，较好地完成了市一届人大各次会议确定的目标任务。

六年来，经济发展迈上了新台阶。坚持把发展作为第一要务，深入开展"项目建设"活动，强力推进工业化、城镇化和农业现代化，加快了经济发展步伐。据统计，去年全市完成生产总值570.8亿元，比2000年增加290亿元，年均增长10%;二、三产业占GDP的比重达68%。项目建设取得实质性突破，特别是累计新上了121个3000万元以上的工业项目，一大批工业项目的建成投运，使限上工业增加值突破了100亿元，比2000年增加66亿元，年均增长17.7%。粮食总产达到622.8万吨，占全省的近1/8，比2000年净增253万吨，年均增长9.1%;新增造林面积120万亩，全市整体实现平原绿化高级达标;畜牧业产值达到140亿元，占农业总产值的46%。完成财政一般预算收入16.2亿元，按可比口径比2000年净增6.6亿元，年均增长19.5%，税收占一般预算收入的比重达70.6%。城镇化率达到20.6%，比2000年提高8.5个百分点。全市综合经济实力明显增强，经济持续、健康、快速发展的内在机制逐步形成，已经步入了发展的快车道。

六年来，基础设施建设实现了重大突破。累计完成全社会固定资产投资726亿元，年均增长23.2%。城市基础设施建设力度加大，中心城区建成区面积由2000年的23.5平方公里扩大到48平方公里，累计新修、改造城市道路38条，铺设供水管网207公里、燃气管道203公里，建成了一大批城市供水、供热、供气、污水处理和社会事业项目，成功创建了省级园林城和卫生城，城市人居环境有了明显改善。农业基础设施建设得到加强，累计新打机井3.7万眼，新增有效灌溉面积186.2万亩、旱保田138.5万亩，治理水土流失481平方公里，解决农村饮水不安全人口45万人。交通事业快速发展，累计投入建设资金157亿元，新增四级以上公路6030公里，其中"村村通"4800公里、高速公路152公里，全市四级以上公路总里程达到12093公里。能源建设取得重大进展，建成了华润、蓝天两大电厂，新增发电装机容量142万千瓦。基础设施的改善，使城乡面貌发生了显著变化，为我们今后的发展奠定了基础，积蓄了后劲，提供了保障。

六年来，改革开放取得了显著成效。国有企业改革稳步推进，全市140户拟改制工业企业有137户完成改制任务;农村税费改革顺利实施，全部免征了农业税，农民负担大大减轻。乡镇机构改革任务顺利完成，撤销合并乡镇24个，分流人员22452人;大力推进行政审批制度改革，共取消、合并、下放行政审批事项1148项;粮食购销企业改制任务全面完成，财税体制改革顺利推进，农村信用社和城市信用社改革取得阶段性成果;交通、城建、水利以及教育、文化、卫生等改革也都取得了重大进展。坚持以东西合作为载体，大力开展招商引资，不断扩大对外开放，累计引进市外资金180多亿元，实际利用境外资金1.45亿美元，年均增长148%;完成出口3.56亿美元，增长44%，促成了香港华润、日本住友、英国爱农、中国昊华、山东鲁洲、中集集团、青岛双星等一批国内外知名企业落户我市，2006年被评为跨国公司眼中最具投资潜力的20个中国城市之一。

六年来，各项社会事业得到了全面发展。坚持统筹经济社会协调发展，切实加大投入，推进了社会事业的全面进步。累计实施科技项目1523项，获省级以上科技进步奖28项，申请国家发明专利310件，发展高新技术企业19家，高新技术产业增加值占限上工业的比重达到8.2%。不断加大对教育的投入，累计改造农村中小学D级危房73.7万平方米，办学条件明显改善，"普九"成果得到巩固;新建高中22所，在校高中生达14.6万人，比2000年增加9万余人;职业教育、成人教育健康发展，黄淮学院申办成功，结束了没有本科院校的历史。全民健身运动广泛开展，竞技水平明显提高，在全省十运会上取得了优异成绩。文学艺术、新闻出版、广播电视、社会科学等社会事业全面进步，文化资源优势得到发挥，共有17件作品获国家级奖项，12件作品获全省"五个一工程"奖;获得国家级、省级非物质文化遗产18项、中国民间文化之乡5个，国家级文物保护单位增加到9处;建成全国、全省文化先进县各2个，省级文化先进乡镇23个。公共卫生、疾病预防和医疗救助体系不断完善，新建疾病控制中心10个，传染病医院和紧急救援中心11个，建设改造乡镇中心卫生院118所，城乡居民看病难、看病贵问题得到缓解;成功战胜了"非典"疫情，艾滋病防治救助取得了明显成效;新型农村合作医疗试点工作稳步推进，试点县参合率达80%。计划生育工作得到加强，人口自然增长率低于全省平均水平。对水污染、大气污染进行了有效治理，环境质量得到明显改善。深入开展拥军优属和军民共建活动，荣获全国"双拥模范城"称号。统计、人事、气象、人防、史志、档案、外事侨务等工作都取得了新的成绩。

六年来，人民生活水平有了明显提高。坚持以人为本，更加关注民生，注重解决关系人民群众切身利益的问题。认真做好就业再就业工作，累计新增城镇就业25.8万人，实现再就业12.8万人，其中"4050"人员3.3万人，城镇登记失业率控制在4%以内。社会保障和社会救助体系不断完善，企业离退休人员"两金"实现了按时足额发放，城镇和农村低保对象做到了应保尽保。全面落实各项惠农政策，种粮直补等七项补贴使农民直接受益7.2亿元;城镇居民人均可支配收入达到7901元，比2000年增加3494元;农民人均纯收入达到2906元，比2000年增加1001元。社会消费品零售总额达187.3亿元，年均增长11.9%;城乡居民储蓄存款余额383.8亿元，比2000年增加236亿元;城镇居民人均居住面积达23.5平方米，比2000年增加8.3平方米。切实让广大人民群众享受到了改革发展的成果。

六年来，社会大局保持了和谐稳定。认真开展保持共产党员先进性和社会主义荣辱观教育活动，切实加强公民思想道德建设，深入开展精神文明创建活动，城乡面貌和干部群众的精神风貌发生了显著变化。自觉接受人大法律监督和政协民主监督，累计办理人大代表建议和政协委员提案1757件，办结率100%。坚持依法治市，深入开展普法教育，"四五"普法任务全面完成， "五五"普法工作扎实推进。作风建设和廉政建设深入开展，部门和行业风气继续好转，反腐败斗争取得了新成效。切实加大对"四乱"的整治力度，坚决打击干扰企业项目施工和生产经营的违法行为，营造了良好的发展环境。认真落实党的民族宗教政策，民族关系更加和谐。深入开展"平安建设"，强化社会治安综合治理，完善社会治安防控体系，严厉打击各种刑事犯罪，社会治安形势良好，近三年连续被评为全省社会治安综合治理和平安建设先进市。认真落实安全生产责任制，及时排查消除各类安全隐患，有效防止了重特大事故的发生。坚持依法治访，认真解决信访突出问题，妥善处置群体性事件，积极化解社会矛盾，全市呈现出人民安居乐业、社会和谐稳定的良好局面。

各位代表，过去的六年，是国民经济健康快速发展，综合经济实力明显增强的六年;是基础设施建设取得重大突破，发展条件和环境不断改善的六年;是三大文明建设共同进步，各项社会事业全面发展的六年;是城乡居民生活水平普遍提高，人民群众得到实惠最多的六年。回顾六年的历程，我们深切地感到，驻马店之所以有今天的大好局面，是省委、省政府和市委正确领导的结果，是全市800多万天中儿女团结奋斗、辛勤努力的结果。在此，我代表市人民政府向工作在各条战线的广大工人、农民、知识分子、干部、驻驻部队指战员、武警官兵和公安民警，向所有关心支持政府工作的各位人大代表、政协委员和民主党派、工商联、无党派人士，表示崇高的敬意和衷心的感谢!

各位代表，过去的六年，我们的确取得了很大的成就，同时也要清醒地看到，在前进道路上还面临着不少困难和问题，主要是:经济总量小，人均水平低，综合实力不强，区域竞争压力较大;经济结构层次较低，增长方式粗放，高新技术产业贡献率不高，工业对经济发展的支撑作用不强，三产发展滞后;农业基础薄弱，产业化水平不高，粮食稳定增产和农民持续增收难度较大;县域经济发展缓慢，县乡财政比较困难，收支矛盾仍然突出;一些关系人民群众切身利益的问题还没有得到很好解决，部分群众的生活还很困难，构建和谐社会的任务非常艰巨;发展环境不够宽松，政府的服务质量有待提高，公务员队伍建设有待加强等等。对于这些问题，各级政府务必给予高度重视，采取有效措施，不断加以解决，决不辜负党和人民的期望和重托!

二、今后五年的总体要求和目标任务

今后五年，是我市深入贯彻科学发展观、加快实现平安崛起的关键时期。当前，我们既面临着东部沿海地区产业加快向内地转移，国家促进中部崛起和我省实施中原崛起战略，以及全面建设社会主义新农村的良好机遇;也面临着全国各地你追我赶、竞相发展的区域竞争压力。面对新的机遇和挑战，我们必须时刻保持清醒的头脑，进一步增强发展的紧迫感和责任感，敏锐把握时代脉搏，深刻认识市情，找准发展定位，凝聚各方力量，争创发展新优势，努力把我市的现代化建设事业不断推向前进。

今后五年政府工作的总体要求是:以邓小平理论和"三个代表"重要思想为指导，以科学发展观统领经济社会全局，坚持把发展作为第一要务，继续深化"三项建设"，牢固树立工业兴市意识，强力推进工业化，加快城镇化和农业现代化，转变增长方式，强化自主创新，推动经济转型，完善体制机制，促进社会和谐，全面增强综合实力、发展活力和区域竞争力，实现经济社会又好又快发展，努力打造实力驻马店、活力驻马店、文明驻马店、和谐驻马店。

今后五年政府工作的主要奋斗目标是:

--经济发展再上新台阶。全市生产总值年均增长11%以上，到2011年接近1000亿元，人均超过1万元;财政一般预算收入年均增长13%，全社会固定资产投资年均增长30%。

--经济结构进一步优化。产业结构形成"二三一"格局，二、三产业比重达到75%以上;支柱产业的支撑带动作用明显增强，非公有制经济快速发展，高新技术产业增加值占GDP的比重不断提高，城镇化率达到30%以上。

--改革开放取得新进展。重要领域和关键环节的改革取得重大进展，经济发展的动力明显增强;开放型经济持续发展，实际利用外资年均增长25%，出口年均增长15%。

--可持续发展能力不断增强。单位生产总值能耗年均下降4%，人口自然增长率控制在6‰以内，努力建设资源节约型和环境友好型社会。

--人民生活水平稳步提高。城镇就业岗位持续增加，社会保障体系比较健全，城镇登记失业率控制在4%以内，社会消费品零售总额年均增长13%，城镇居民人均可支配收入、农民人均纯收入年均增长8%和5%左右。

--和谐社会建设取得显著成效。科技教育事业全面发展，卫生事业不断进步，文化事业进一步繁荣，民主法制、精神文明和平安建设取得新进展。

实现上述目标，我们必须抓住新一轮战略机遇期，进一步理清发展思路，突出工作重点，确保各项工作有所创新、取得突破、得到全面落实。在工作指导上要着力把握好以下几点:一是牢固树立科学的发展观，始终把发展作为第一要务，排除各种干扰，抓着一切机遇，千方百计调动各方面的积极性和创造性，聚精会神搞建设，一心一意谋发展，努力实现发展的新突破。二是继续深化"三项建设"，紧紧围绕驻马店平安崛起目标，以项目建设为载体，强力推进新型工业化，努力加快城镇化和农业现代化，使"三化"互促互动，不断扩大经济总量，进一步壮大经济实力。三是切实加大结构调整力度，以科技创新为动力，以市场为导向，继续优化产业结构、城乡结构、所有制结构，大力推进资源节约和综合利用，高度重视环境保护，切实转变经济增长方式，努力实现经济发展与人口、资源、环境相协调，不断提高经济增长的质量和效益，继续增强可持续发展的能力。四是进一步解放思想，转变观念，与时俱进，坚决冲破一切妨碍发展的思想观念，坚决改变一切束缚发展的做法和规定，坚决革除一切影响发展的体制机制障碍，积极推进重要领域和关键环节的改革，坚定不移地以东西合作为载体，加大招商引资力度，努力扩大对外开放，不断为经济社会发展注入新的生机与活力。五是牢固树立以人为本的执政理念，更加关注民生，切实把实现和维护人民群众的根本利益作为一切工作的出发点、落脚点，认真解决人民群众关心的热点难点问题，实实在在地为群众办实事、办好事，让群众充分享受到改革发展的成果。六是统筹经济社会协调发展，进一步强化政府公共服务和社会管理职能，不断加大对科技、教育、文化、卫生等领域的投入，大力发展各项社会事业，促进经济与社会的相互适应、良性互动，努力实现人的全面发展和社会的全面进步。七是正确处理改革、发展、稳定的关系，始终把建设平安驻马店作为加快发展的重要保障，发扬民主，加强法治，积极探索建立维护社会稳定的长效机制，在稳定中求发展，以发展促稳定。

各位代表，驻马店资源丰富，区位较好，发展潜力巨大。我们坚信，有省委、省政府和市委的坚强领导，有近年来打下的良好基础，只要我们充分调动一切积极因素，抢抓机遇，加快发展，就一定能够开创我市改革开放和现代化建设的新局面，为实现驻马店平安崛起做出新的更大的贡献!

三、2007年工作的主要目标和任务

今年是"十一五"规划的第二年，保持全市经济社会发展的良好势头，对整个"十一五"的发展至关重要。今年我市经济社会发展的主要预期目标是:全市生产总值增长11%以上，限上工业增加值增长20%以上，全社会固定资产投资增长30%，社会消费品零售总额增长14%以上，财政一般预算收入增长13%以上，城镇居民人均可支配收入增长8%，农民人均纯收入增长5%，城镇登记失业率控制在4%以内，单位生产总值能耗降低4%左右，人口自然增长率控制在6‰以内。实现上述目标，应重点抓好以下八个方面的工作:

(一)加大项目建设力度，进一步壮大工业经济。今年初步安排重点建设项目148个，年度计划投资101.9亿元。其中计划竣工项目84个，年度投资48.6亿元;续建项目21个，年度投资8.4亿元;计划新开工项目37个，年度投资42.1亿元;前期项目6个，年度投资2.7亿元。对于这些重点项目，要进一步完善工作责任制，切实解决好项目管理、资金落实、征地拆迁、建设环境等问题，确保完成年度目标任务。突出抓好工业项目建设。今年初步安排重点工业项目110个，年度计划投资46.1亿元。要重点抓好天方药业洁霉素、蓝天集团燃气电厂、吴桂桥煤矿、红星网业、豪美纸业、双星轮胎等70个计划竣工项目，确保按期竣工投产;抓好中集华骏10万吨铸件、众品食业、天中粉业等10个续建项目，确保完成年度投资计划;抓好蓝天集团20万吨烯烃、顺达化工20万吨醋酸、白云纸业二期、豫龙水泥二期、中集华骏年产5000辆罐式车、置地太阳能热水器、豫美石材、徐福记食品等26个新开工项目，确保按期开工建设。抓好市热电厂2×30万千瓦热电联产、华润古城2×100万千瓦燃煤电厂、昊华骏化合成氨原料路线改造、中原鞋都龙胜工业园等项目的前期工作，并围绕我市产业和资源优势，积极论证储备一批大项目，争取更多的项目纳入国家和省计划。积极推进工业结构优化升级。加快推进醋酸、甲醇、烯烃等重大项目建设，着力打造煤化工基地;支持天方药业、中泰药业等系列产品的开发生产，不断壮大医药产业;大力发展农畜产品加工、建材和机械制造业，努力形成规模优势。支持50户重点企业引进战略合作伙伴，加强企业并购、联合重组和资本运作，促使企业做大做强。继续抓好工业园区建设，完善基础设施和规划布局，大力推广标准厂房建设，努力推进工业集聚发展、基地化发展。着力提高企业自主创新能力。支持企业加大技改力度，推进传统产业、产品升级换代。不断加大科技投入，支持骨干企业建立研发中心，引导企业加强科技创新和科技合作，积极引进和培养技术人才和管理人才，加快培育新兴产业。积极推广应用先进节能技术，加快淘汰落后工艺、设备和生产能力，促进节能降耗。加强对工业运行的协调指导。引导企业加强内部管理，强化市场营销，提高经济效益。搞好工业运行的监测分析，及时解决工业运行中的问题。继续加大环境整治力度，切实保护企业的合法权益。积极做好企业推荐和项目推介，促进银企合作。

(二)坚持以发展农村经济为重点，扎实推进新农村建设。继续把"三农"工作作为重中之重，促进农村全面发展。认真落实支农惠农政策。稳定对种粮农民的直接补贴规模，扩大良种补贴和农机具购置补贴范围，加大农业生产资料综合补贴力度，落实好农村劳动力转移培训等补贴政策。继续实行粮食最低收购价政策，保持粮食和农业生产资料价格基本稳定。大力发展现代农业。加快西平、遂平、正阳、上蔡、汝南、平舆、新蔡7县国家优质小麦基地建设，稳定粮食种植面积，继续提高粮食综合生产能力。大力发展高效经济作物和无公害农产品，不断扩大蔬菜、水产、花生、食用菌、花木等特色农产品的生产规模。支持正阳、新蔡、泌阳、西平、遂平5个省定畜牧业重点县的发展，突出抓好养殖小区建设，全面建设县级动物防疫体系，继续提升畜牧业的发展水平。积极发展速生丰产林，努力提高林业生产效益。加强农业科技和标准化工作，全面推进农业科技成果进村入户。努力推进农业产业化。要充分发挥我市粮油、畜禽和林业资源优势，大力发展精深加工业，重点抓好鲁洲生物、天歌鹅业、丰盛粮油等73家龙头企业，不断扩大生产规模，拉长产业链条，力争农畜产品加工业销售收入达到200亿元。进一步完善龙头企业与农户的利益联结机制，大力发展专业合作和中介组织，继续提升农业生产的组织化程度。进一步改善农村生产生活条件。努力实现所有行政村通水泥(油)路，90%以上行政村通公交车，解决18万农村居民安全饮水问题，新增农村沼气用户6万户。切实抓好农村中小学校舍维修改造和课桌凳的更新配置，全部完成农村中小学现代远程教育工程建设，完成310个自然村通广播电视，并在200个行政村实施农村电影放映工程。加强新农村建设规划，继续抓好102个新农村建设试点工作，逐步培育一批新农村建设典型。大力发展劳务经济。完成农村劳动力引导性培训10万人、技能性培训8万人，全市劳动力转移保持在200万人次以上。加大扶贫开发整村推进力度，确保7万农村贫困人口脱贫和解决温饱。

(三)加快城镇化进程，统筹城乡协调发展。坚持以创建活动为载体，加强规划、建设和管理，努力提高城镇化水平。着力抓好中心城区建设。全面实施行政新区搬迁，完善基础设施和服务功能，提高新区集聚水平。加大旧城改造力度，抓好背街小巷的整治，启动4个"城中村"改造项目，完成拆迁改造面积26万平方米。建设经济适用房10万平方米、廉租住房1万平方米。抓好城市游园建设，实施道路街景改造，进一步改善人居环境。继续深入开展"四城联创"活动，加强城市管理，加大执法力度，巩固创建成果，确保创建目标如期实现。尽快实施驿城大道南北延伸工程，搞好工业布局规划，引导项目聚焦，逐步形成连接两县一区的工业走廊，加快遂平、确山和中心城区的一体化进程。加快县城和小城镇建设。立足现有基础，发挥比较优势，加大投入，完善基础设施，加快旧城改造步伐，加强城镇综合管理，进一步改变城镇面貌。大力培育支柱产业、特色产业，壮大城镇经济，增强城镇的吸纳集聚能力。创新城镇发展的体制机制，消除发展障碍，做好撤乡设镇、撤镇设办和城镇人口统计工作，努力提高城镇化水平。继续抓好第三产业。加强物流基础设施建设，积极发展大型物流企业。加快物流市场体系建设，积极培育壮大一批大型综合、专业批发市场。抓好"万村千乡"试点工程建设，健全农产品进城、工业品下乡的流通体系。进一步落实政策，加大投入，大力发展房地产业，不断完善住房市场和服务。积极发展金融、信息咨询、中介服务、社区服务等现代服务业。抓好旅游精品景区建设，整合旅游资源，完善基础设施，加强宣传推介，推动旅游产业快速发展。大力发展县域经济。抓好各项政策落实，加强分类指导，加压驱动，支持各地以工业为主导，以民营经济为主体，以招商引资为手段，培育壮大特色产业，加快发展步伐，不断增强县域经济的综合实力。

(四)加快基础设施建设，继续改善发展条件。进一步优化投资结构，抓好一批重大基础设施项目建设，增强对发展的支撑和拉动作用。加强水利基础设施建设。加快大、小洪河治理工程和老王坡、杨庄滞洪区安全建设，开工建设薄山、宋家场等水库除险加固工程，抓好板桥、薄山大型灌区续建配套及节水改造项目建设，加强西部山区抗旱水源和小流域治理工程建设。积极做好宿鸭湖水库除险加固和灌区配套、平原洼地治理等重点项目的前期工作，力争纳入国家投资计划。抓好中低产田改造、基本农田保护、节水灌溉建设，完成30万亩中低产田改造和40平方公里小流域治理任务。加强交通基础设施建设。加快新蔡至驻马店、驻马店至泌阳、平舆至正阳3条高速公路和张南路、平长路、君二路、商桐路等10条干线公路项目进度，确保年底前建成通车。抓好焦桐高速公路泌阳段、安信快速通道驻马店段、化庄(省界)至新蔡高速公路、郑州武汉客运铁路专线等项目的前期工作。全面完成672个行政村"村村通"和62个农村客运站建设任务，加强电力基础设施建设。切实抓好嵖岈山500千伏变电站，新蔡、西平等3个220千伏和正阳彭桥、确山新安店等7个110千伏输变电站工程，加快城乡电网建设与改造步伐，进一步完善电网结构，全面完成"户户通电"工程。加强城市基础设施建设。抓好市区南海路南段、淮河大道西段、驿城大道、中华大道西段等13条道路建设，加快通达小区、森林半岛、万家花园等房地产项目建设，开工建设市体育中心、妇女儿童活动中心、工人文化活动中心、博物馆、群艺馆、福利院，加快市广电中心和图书馆建设进度，建成市科技馆，完成9个县城污水处理厂和垃圾处理场的建设任务。

(五)强力推进改革开放，努力增强发展活力。推进国有企业改革。鼓励和引导国有企业引进战略合作伙伴，加快企业股份制改造和重组，完善法人治理结构，基本实现产权多元化。继续推进商贸、粮食、供销等非工业企业改革，加快市直单位所属企业改革。大力发展非公有制经济。认真落实各项政策措施，鼓励和引导非公有制资本进入现代服务业、高新技术产业和社会事业领域。构建面向中小企业的公共服务平台，加强信用担保体系和信用制度建设，着力解决中小企业融资难问题。推进农村综合改革。深化和完善乡镇机构改革，确保乡镇机构、编制和财政供养人员只减不增。完善"乡财县管"制度，增强基层财政保障能力。落实教育经费保障机制，确保各级财政应承担的经费足额到位。深化农村信用社改革，全面开展林权制度改革，认真落实减轻农民负担的各项政策，防止农民负担反弹。加强财税收支管理。加快部门预算改革步伐，全面实现部门预算编制信息化管理。进一步扩大政府采购和政府投资评审规模及范围。加强和完善税源管理机制，提高税收征管质量。严格实行各项政府非税收入的"收支两条线"管理。加快发展外向型经济。筹备组织好2007年全国东西合作经贸洽谈会，积极参加中部投资贸易博览会、豫港投资贸易洽谈会等大型贸易招商活动，多渠道引进项目和资金。围绕资源特点、优势产业、现代服务业、基础设施和国有企业产权转让等，搞好项目推介，有效开展招商活动。抓好利用外资工作，努力提高利用外资规模和水平。继续做好签约项目的跟踪落实，提高项目履约率和资金到位率。认真落实招商引资责任制，加大考评和奖罚力度，优化发展环境，推进招商引资再上新台阶。继续抓好优势农畜产品出口，不断扩大工业品出口规模，切实加强对外劳务合作，努力把我市对外开放提高到一个新的水平。

(六)统筹经济社会发展，推进社会事业全面进步。全面发展科技事业。切实围绕工农业生产薄弱环节，组织实施一批科技攻关项目。进一步优化科技资源配置，加强科技队伍建设，完善科技服务体系，突出抓好农村科技服务社和企业技术中心建设，促进科技成果的转化。继续加强同高等院校和科研院所的交流与合作，引进、吸收和消化一批科技成果，不断提高科技对经济的支撑能力。优先发展教育事业。进一步完善投入机制，逐步增强各级政府义务教育保障能力。全部免除农村义务教育阶段学杂费，继续对困难家庭学生免费提供课本并补助寄宿生生活费。推进农村教育资源整合，建成一批寄宿制小学。多策并举，加强城镇基础教育，扩大高中阶段教育规模，大力发展职业教育，鼓励引导民办教育发展，积极支持黄淮学院建设，加快筹建驻马店职业技术学院。大力发展文化体育事业。加大对公益性文化事业投入，抓好文化基础设施建设，积极开展广场文化、社区文化及文化下乡活动，逐步健全城乡公共文化服务体系。加强文化市场和网络文化管理。抓好文化资源整合，深化文化领域改革，推进文化产业发展。实施文化精品工程，繁荣广播电视、文学艺术、社会科学、新闻出版事业。广泛开展全民健身运动，努力提高竞技体育水平。加快发展医疗卫生等事业。抓好2所县级医院和21所乡镇卫生院改造，开工建设市中心医院病房大楼和河南友谊肿瘤医院。加强新型农村合作医疗试点工作，将试点范围扩大到5个县。规范医疗器械和药品招标采购，加强医德医风建设，坚决治理医疗乱收费，切实降低药品价格和医疗收费，认真解决群众看病难、看病贵问题。继续做好艾滋病防治工作，不断提高防治和帮扶工作水平。加强国防教育，做好国防动员、人民防空和民兵预备役工作，落实优抚安置政策，深入开展双拥共建活动，巩固和发展军政军民关系。依法保护妇女、儿童、未成年人和残疾人的合法权益。同时，做好统计、人事、外事侨务、史志、档案、人防、气象等工作。落实可持续发展战略。坚持计划生育国策地位不动摇，切实加强人口计生工作，统筹解决出生人口性别比偏高等问题，继续稳定低生育水平。强化对水、森林、土地、矿产等自然资源的生态保护，全面推进节水型社会建设，提高水资源利用效率。加强退耕还林、森林生态工程、湿地保护和绿色工程建设，确保完成成片造林11万亩，新建通道绿化800公里。落实耕地保护措施，加强土地"三项整治"，促进土地集约利用。推进矿产资源整合，不断提高矿产资源开发利用水平。全面落实环境影响评价、污染物排放总量控制等制度，继续加强对洪河、汝河、黑河流域的水污染综合整治，巩固水污染防治成果。对重点企业实施污染物排放控制，确保化学需氧量削减9%、二氧化硫排放量削减5%。

(七)切实解决民生问题，促进社会和谐稳定。坚持以人为本，关注民生，采取多项措施，推进和谐社会建设。切实做好就业再就业工作。大力发展就业吸纳能力强的劳动密集型产业，完善和落实小额贷款、税费减免政策，加强职业技能和创业培训，多渠道开发就业岗位。重点帮助"4050"人员等困难群体就业，确保全年城镇新增就业4.2万人，消除零就业家庭200户以上，实现下岗失业人员再就业1.2万人，其中"4050"人员4000人。进一步完善社会保障体系。积极扩大城镇社会保险覆盖面，努力把养老、医疗、失业、工伤和生育五项社会保险覆盖到各类企业。完善城市居民最低生活保障制度，实现动态管理下的应保尽保。完善农村低保政策，最低补差标准提高到每人每月不低于30元，覆盖24万人左右。加快敬老院建设，农村五保户集中供养率达到30%。大力发展以扶老、助残、救孤、济困为重点的社会福利和慈善事业。加强精神文明和民主法制建设。深化爱国主义、集体主义和社会主义教育，广泛开展文明行业、文明单位、文明村户创建活动，深入开展文化、科技、卫生"三下乡"和科教、文体、法律、卫生进社区活动，在全社会树立知荣辱、讲正气、树新风、促和谐的新风尚。认真执行人大及其常委会的决议、决定，支持人大代表依法行使职权。自觉接受政协的民主监督，认真办理人大代表建议和政协提案。广泛听取各民主党派、工商联和无党派人士的意见，充分发挥工会、共青团、妇联等人民团体的桥梁纽带作用。全面贯彻党的民族宗教政策，继续做好民族宗教工作。大力推进政务、厂务、村务公开，保证人民群众的知情权、参与权和监督权。深入开展普法教育，加强法制宣传，全面提高公民法律素质，积极推进依法治市进程。切实做好社会稳定工作。继续深化"平安建设"，健全社会治安防控体系，切实抓好命案侦破、打黑除恶等专项斗争，严厉打击各种违法犯罪活动。认真落实安全生产责任制，切实抓好重点领域和行业的专项整治，坚决遏制重特大事故发生。大力整顿和规范市场经济秩序，加强食品药品安全监管，严厉打击制假售假行为。进一步加强信访工作，依照法律和政策及时解决群众反映的问题，积极化解矛盾和纠纷，确保社会大局的稳定。

(八)加强政府自身建设，努力提高施政能力。全面完成今年的各项目标任务，必须切实加强政府自身建设，不断提高政府的管理水平和施政能力。加快服务型政府建设。进一步把政府职能转变到为市场主体提供优质服务和创造良好发展环境上来。大力推进政务公开，在公共服务部门全面实行办事公开制度，广泛接受群众的监督。积极推进电子政务建设，完善行政服务大厅的功能，进一步规范和减少行政审批，简化办事程序，切实履行服务承诺，更好地为基层、企业和公众服务。努力提高管理水平。完善项目建设审批程序和监管机制，加强财政收支管理，强化审计监督，真正把有限的资金用在刀刃上。加强国有资产管理，严格规范国有资产处置行为，确保国有资产保值增值。严格控制公务消费，规范公务接待行为，降低行政成本。加强应急管理体系建设，提高预防和处置突发公共事件的能力。加强对重点工作、重大项目的跟踪监督，健全目标管理制度、督查制度和考核制度，确保各项工作落到实处。全面推进依法行政。认真落实监督法、行政许可法，严格按照法定权限和程序行使权力、履行职责。加强行政法制建设，强化行政执法监督，搞好行政复议。加强审计监督、效能监察，做到有权必有责、用权受监督、违法要追究。在土地征用、城市拆迁、企业改制、环境整治等方面，严格按法定程序办事，坚决纠正损害群众利益的行为。加强政风建设。切实增强公仆意识，牢固树立正确的政绩观，积极推进决策的科学化、民主化，大兴求真务实之风，多做打基础、管长远的事情，所有工作都要经得起实践的检验。大力精简会议文件，切实改进会风文风，进一步清理评比达标活动，力戒形式主义和官僚主义。积极开展"讲正气、树新风"活动，加强对政府工作人员的教育、管理和监督，深入开展廉政建设和反腐败斗争，努力建设一支高素质的公务员队伍，为人民服务、对人民负责、让人民满意。

各位代表!

驻马店加快发展的蓝图已经绘就,任务艰巨，责任重大。让我们以邓小平理论和"三个代表"重要思想为指导，全面落实科学发展观，团结奋进，开拓创新，扎实工作，不断开创我市经济社会发展的新局面，为全面建设小康社会和实现驻马店平安崛起而努力奋斗!
